# Institutional quality and COVID-19 vaccination: does decentralization matter?

**DOI:** 10.1007/s12076-023-00326-y

**Published:** 2023-02-25

**Authors:** Chiara F. Del Bo

**Affiliations:** Department of Economics, Management and Quantitative Methods, Università degli Studi di Milano, Milan, Italy

**Keywords:** Institutional quality, Federalism, COVID-19 vaccination, Public health policy, E65, H77, O50

## Abstract

Vaccination campaigns are one of the factors that can help mitigate the adverse effects of viral pandemics. The aim of this paper is to understand the institutional factors that are associated with a higher success rate, measured by the percentage of vaccinated population against COVID-19 across countries. Along with supply side determinants, institutional factors, related, at the national level, to the organization of the healthcare sector, governance and organization of the State and social capital, and, at the subnational level related to the authority and autonomy of lower tiers of government, are important correlates of successful vaccination campaigns, suggesting potential areas of public policy interventions.

## Introduction

While the current COVID-19 pandemic is still ongoing, researchers warn against the possibility of new viral outbreaks (Carlson et al. 2022) thus suggesting the need to understand how to boost our ability to respond effectively. Vaccination campaigns are one instrument that can help mitigate the adverse effects of pandemics. The aim of this paper is to understand which institutional factors, both at the national and the subnational level, are associated with a higher success rate of a vaccination campaign, measured by the percentage of vaccinated population against COVID-19 across countries. Singling out the institutional aspects related to a successful vaccination rollout may suggest areas for policy intervention aimed at strengthening factors that will, in the event of future pandemics, contribute to curbing the negative consequences associated with them. Understanding the role of decentralization and subnational authority is also useful in light of other global challenges, which require the intervention of different government tiers, such as the climate change crisis.

## Theoretical background

Immunization programs are a key factor of a country’s public health strategy. A strand of the public health literature has thus explored whether institutional covariates are correlated with successful vaccination campaigns and identified the main supply and demand side factors associated with vaccine uptake from the population and, consequently, the overall rollout.

A set of papers have analyzed the interplay between vaccination rates and supply side factors, including the logistics of an immunization campaign (see Duijzer et al. 2018 for a review). Zaffran et al. (2013) examine vaccine supply chains, and stress how with more sophisticated vaccines, such as the ones used against COVID-19, logistical aspects become increasingly important, as does the interplay between national and subnational authorities in procuring, delivering and administering the doses to the population.

Other papers have focused instead on demand side factors, examining the determinants of vaccine hesitancy and the role of individual factors influencing uptake. Donkers et al. (2015) show how the perceived seriousness of Mumps increases vaccination rates among Dutch university students. Similarly, in the context of the current pandemic, Viswanath et al. (2021) show how individual perception of risk of becoming infected or by proximity with casualties from the disease, decreases vaccine hesitancy.

Another strand of research has examined the role of institutional variables, suggesting how these might impact both the supply of vaccines and also influence vaccine hesitancy among the population. With respect to the former, Tatar et al. (2021) stress the importance of good governance and government effectiveness on vaccine rollout while Farzanegan and Hofmann (2021) document how countries with high levels of corruption have been less successful in vaccinating their populations.

Considering instead the latter, Jelnov and Jelnov (2022) show how a corrupt government is perceived by its citizens as more likely to promote an unhealthy vaccine, thus lowering trust and increasing vaccine hesitancy.

The role of national versus sub-national decision making level has been considered for vaccines in general (see e.g. Guyer et al. 2000) and in the context of the immunization response to the current pandemic (Goel and Nelson 2021). Both papers examine the US context, where the main issue is coordination, or lack of, between the federal and state level, along with the degree of centralization of public health authorities.

This set of findings thus forms the foundation for the present empirical work, which can be organized around a research question and a set of research hypotheses.

### **Research Question**

What are the main covariates of the success rate of a vaccination campaign? Are institutional factors among these?

### **Hypothesis 1**

Does the relationship between covariates and the success rate of a vaccination campaign differ at different stages of the rollout?

### **Hypothesis 2**

Does the relationship between institutional factors and success rate of a vaccination campaign differ if the former are at the national or subnational level?

## Empirical model and data

The dependent variable is the percentage of vaccinated people at different stages of the process: people who have received at least one dose (*Vaccinated*); those that have completed the official vaccination program, which differs according to the type of vaccine used and which, in several countries, has been made, in some form, mandatory (*Fully vaccinated*); and those with additional booster doses, usually left to the discretion of citizens even in countries with mandatory programs (*Additional*).

Potential determinants are divided between baseline and institutional factors. The baseline specification includes supply side factors (number of types of vaccines available in the country throughout the vaccination campaign-*Number of vaccine types*- and a dummy variable indicating if the first vaccine used was of the MRNA technology or not- *First vaccine-MRNA*); the severity of the pandemic situation as measured by cumulated COVID-19 related deaths as of July 2020, corresponding to the first wave of the pandemic-*COVID deaths*; the percentage of population living in cities- *Urban population (%)* and a dummy variable indicating whether the country is organized as a federation or not- *Federalist country*.

Country-wide external, institutional conditions may influence the success of vaccination campaigns. There are both supply and demand side arguments. On the supply side, the organization of the healthcare sector is related to the availability and distribution of vaccines, while the organization of the State as unitary or federal may influence the logistics of procuring and distributing vaccines. The overall governance, organization and effectiveness of the public sector may work similarly. On the demand side, trust in the authority providing/imposing the vaccination program may enhance citizens’ willingness to enroll in it, as will the general levels of positive social capital. Institutional variables are thus related to the organization and access to the healthcare system, measured by the Universal Health Coverage index (*UHC*); measures of the institutional strength and organization of states (*Government Effectiveness* and *State Capacity Index*); measures of social capital (*Trust in national government* and *Index of participation*) and the *Index of democratization*, which combines information on electoral success of smaller parties with the voting turnout in elections, thus capturing the quality of the democratic system.

Another important institutional feature that might be associated with the vaccination roll-out is related to subnational (i.e. regional) governments’ autonomy. To this end, a measure of regional authority (*Regional authority index*) and one of its components, namely the level of regional autonomy in terms of policy formulation (*Policy autonomy*) are considered.

**Fig. 1:**
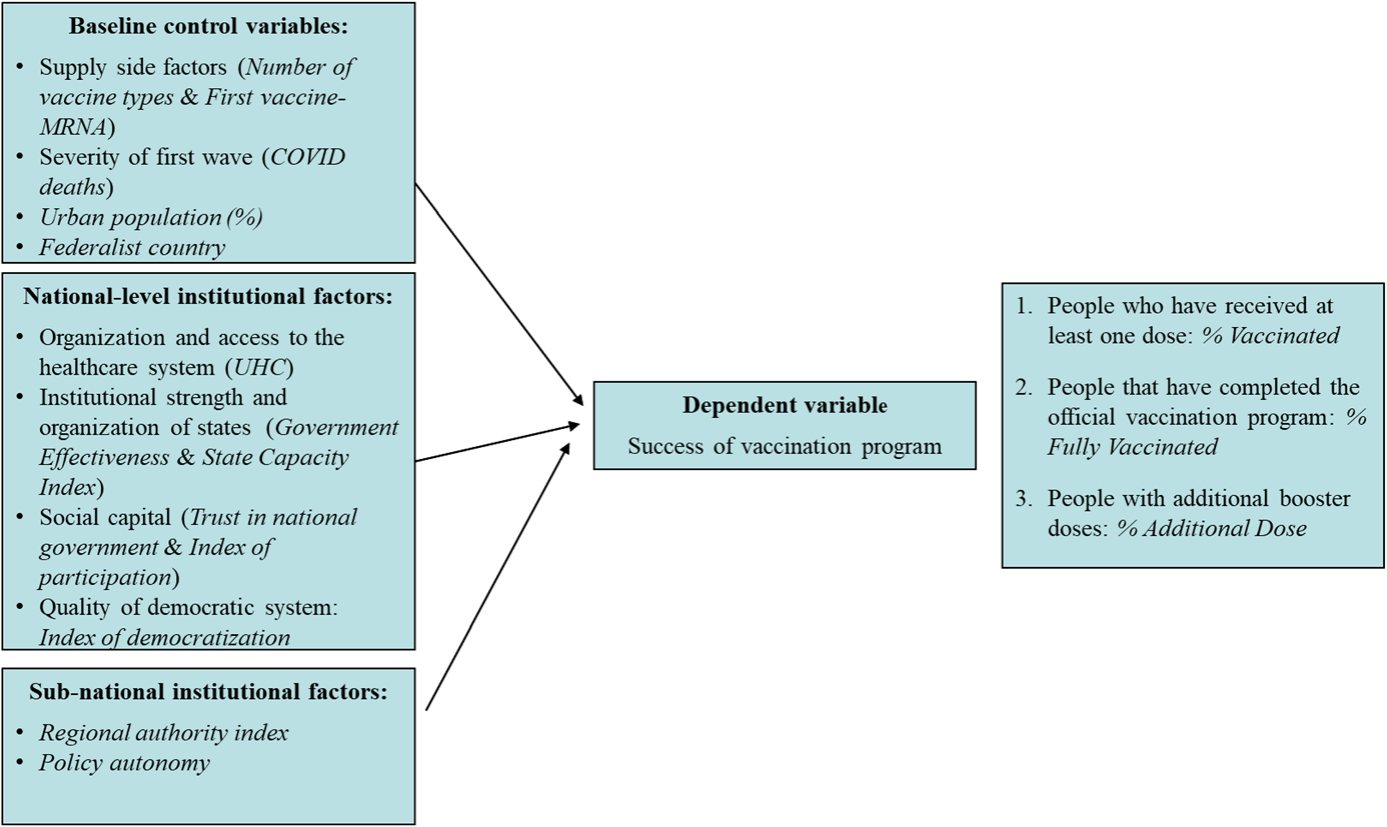
The empirical model

Figure 1 summarizes the empirical model, based on the Research Question and Hypotheses set forth in Sect. 2.

Table 7 provides information on data, sources and variables. The dataset comprises 86 countries (Table 8), although data availability of regressors causes, in some specifications, a decrease in observations.

## Results and discussion

**Fig. 2:**
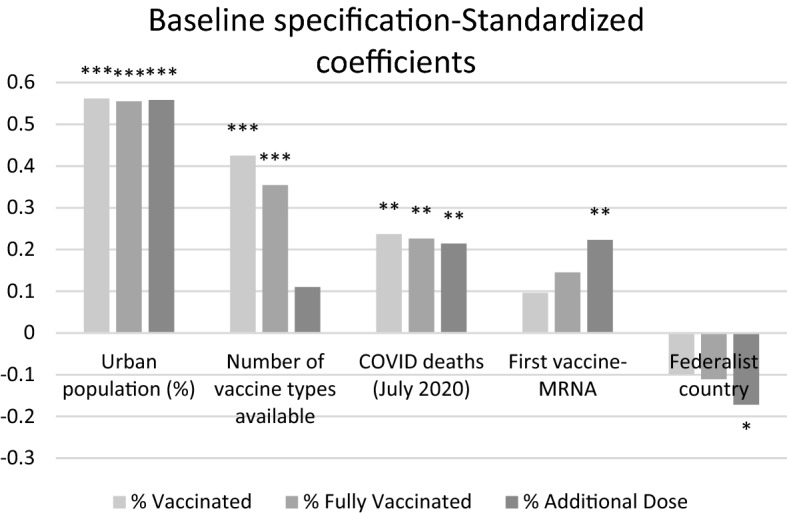
Standardized coefficients of baseline specification

**Fig. 3:**
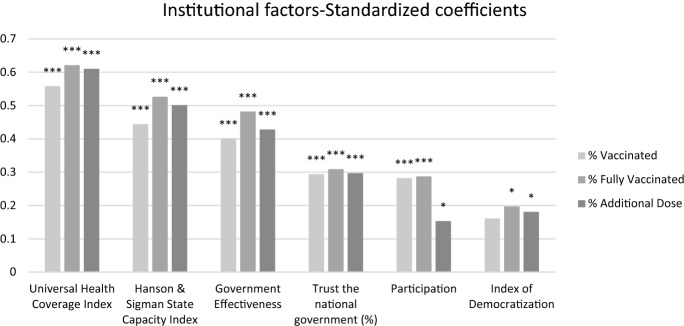
Standardized coefficients of national institutional factors

In Sect. 4.1, results for the baseline specification are presented (Table 1), with a discussion of the magnitude of standardized coefficients (Fig. 2). Section 4.2 focuses on institutional factors, first taking into account country-level institutions (Tables 2, 3, 4; Fig. 2), then considering more in depth the extent of subnational autonomy from the central government (Table 5, Fig. 3).

**Table 1:** Baseline specification

Dep. var.	% Vaccinated	% Fully vaccinated	% Additional dose
	(1)	(2)	(3)
COVID deaths	0.000265**	0.000252***	0.000256***
	[0.000101]	[0.0000927]	[0.0000957]
Number of vaccine types	0.0513***	0.0429***	0.0150*
	[0.0122]	[0.0117]	[0.00862]
First vaccine-MRNA	0.0395	0.0595	0.0994**
	[0.0403]	[0.0411]	[0.0455]
Urban population (%)	0.00671***	0.00665***	0.00718***
	[0.00131]	[0.00129]	[0.00151]
Federalist country	− 0.0479	− 0.0539	− 0.0889*
	[0.0452]	[0.0472]	[0.0493]
Constant	− 0.0669	− 0.123	− 0.391***
	[0.161]	[0.148]	[0.119]
Observations	86	86	84
R-squared	0.467	0.464	0.537

Macroregional effects included. Robust standard errors in brackets

*p < 0.1, **p < 0.05, ***p < 0.01

### Baseline

In the baseline specification, the focus is on the main determinants of the percentage of vaccinated people at different stages of the process (columns1-3, Table 1). *COVID deaths*, which accounts for the cumulative death doll of the first wave, in June 2020 (Del Bo 2021), is positively correlated with the percentage of vaccinated population, irrespective of the stage in the process, in line with previous literature showing how increased perceived risk reduces vaccine hesitancy. Countries which have experienced a higher death toll from the pandemic in the initial phase, when vaccinations were not yet available, have been motivated to speed up the rollout and their population is probably more willing to accept and request vaccines, including boosters.

Similarly, *Urban population (%)* is positively related to the vaccination program in all its stages. Non mutually exclusive explanations are the following. First, urban density might have exacerbated the awareness of the severity of the pandemic and of the importance of vaccination due to the higher education urban rates (Berry and Glaeser 2005).^1^ Second, urban density is associated with higher transmission rates (Gerritse 2020; Rodríguez-Pose and Burlina 2021), possibly making the population more willing to adhere to the vaccination program. Also, from a supply side perspective, urban density may be linked to logistical factors that make the distribution of vaccines easier than in dispersed, rural areas. Finally, the percentage of urban population is a proxy for the density of demand, and the delivery of vaccines may be less costly if demand is spatially concentrated, as the literature on local public services suggests (Hortas-Rico and Solé-Ollé 2010).

Continuing with supply side arguments (Goel and Nelson 2021), *Number of vaccine types* is positively related to the percentage of vaccinated people with the main protocol (*Vaccinated* and *Fully vaccinated*) while not to booster doses. Along the same lines, the use of MRNA vaccines at the beginning is related to a higher percentage of population with additional doses but not to the regular program. A greater availability of vaccine types may increase the rollout in the main program, due to logistical aspects and by making the country less vulnerable to shortages from producers, but the lack of an association with additional doses suggests that, in this stage, other factors are at play. This is corroborated by the finding related to the type of the first vaccine, with MRNA vaccines (i.e. the most effective, Rotshild et al. 2021) positively associated with higher percentage of people with booster doses, suggesting that higher efficacy might be related to higher acceptance and trust in additional doses beyond the official and, in some cases, required protocol. This result helps answer Hypothesis 1, suggesting that indeed determinants differ across stages of the vaccination process.

A federal state, compared to a unitary- one, might be better organized in procuring and administering vaccines, due, for example, to logistical reasons (Greer et al. 2022; OECD 2022). This claim is not supported by the data used here, since *Federalist* is not statistically distinguishable from zero for the official protocol and is negatively associated with additional doses.

Focusing on the magnitude of coefficients, standardized beta coefficient are computed for the baseline specification and presented in Fig. 12. From inspection of Fig. 2, *Urban population (%)* is the most important determinant of the success of the campaign in terms of vaccinated population, across all stages. The second most important factor, but only for the official program, is the availability of different types of vaccines. The severity of the pandemic in the first wave is also positively correlated with administered doses at all stages. Relevant factors for additional doses only are if the first vaccine used was MRNA and whether the country is a federation (negative estimated coefficient).

### Institutional factors at the national level

In Tables 2, 3 and 4 (dependent variables *Vaccinated*, *Fully vaccinated* and *Additional dose*), institutional factors at the national level are included, thus providing empirical evidence useful to answer the paper’s main research question. Main results for baseline variables are confirmed.

With respect to the percentage of population with at least one dose, all institutional variables are positively related to the vaccination rate and statistically distinguishable from zero, with the exception of *Index of democratization*. Similar considerations for *Fully vaccinated* and *Additional dose*, with *Index of democratization* now statistically different from zero.

Looking at the magnitude of estimated coefficients, in Fig. 3 standardized coefficients are presented in decreasing order of magnitude. As a general comment, higher values are found in relation to the dependent variable *Fully vaccinated*.

The largest value, for all dependent variables, is associated with *UHC*, suggesting the importance of access to quality and affordable healthcare (Van de Pas et al. 2022): a one standard deviation of *UHC* is associated with a 0.558 standard deviation of vaccinated population. *UHC* is a standardized index measuring the proportion of population that can access essential quality healthcare without using a large percentage of household income on health. In the context of the current pandemic, a high coverage health system is associated with a more effective vaccination campaign. *State Capacity* is based on executive, coercive and administrative capacity (Tevdovski et al. 2022). This is the institutional factor with the second highest estimated coefficient. Countries with higher state capacity have a higher proportion of vaccinated people at all stages of the process. Similarly, *Government effectiveness* is the third most important factor. Taken together these results related to three supply side factors, suggest that stronger, more effective states providing quality and affordable healthcare, have the power, organization and resources necessary to proceed with the vaccination program.

Considering measures of social capital (Lazarus et al. 2021), *Trust in national government* presents very similar values of estimated coefficients at the different stages of the vaccination program, and has values very similar to *Participation*.

The smallest values are associated with *Index of democratization*, suggesting that this institutional aspect might be less relevant for a successful vaccination campaign.

Overall, institutional variables are significantly related to the success of the vaccination campaign, while this relationship does not seem to change much according to the phase of the process, suggesting that, at least for these types of variables, Hypothesis 1 does not hold.

**Table 2:** Determinants of % of population with at least one dose of vaccine

Dep. var. % vaccinated	(1)	(2)	(3)	(4)	(5)	(6)
COVID deaths (July 2020)	0.000163*	0.000231**	0.000220**	0.000196**	0.000261**	0.000206**
	[0.0000868]	[0.0000940]	[0.0000953]	[0.0000957]	[0.000101]	[0.000101]
Number of vaccine types	0.0531***	0.0495***	0.0492***	0.0494***	0.0462***	0.0499***
	[0.0107]	[0.0109]	[0.0115]	[0.0101]	[0.0116]	[0.0115]
First vaccine-MRNA	− 0.0464	− 0.02	0.0169	0.0186	0.0518	− 0.0146
	[0.0370]	[0.0412]	[0.0376]	[0.0372]	[0.0393]	[0.0396]
Urban population (%)	0.00410***	0.00526***	0.00655***	0.00612***	0.00631***	0.00517***
	[0.00111]	[0.00102]	[0.00127]	[0.00121]	[0.00143]	[0.00124]
Federalist	− 0.0381	− 0.0498	− 0.055	− 0.0387	− 0.0362	− 0.0297
	[0.0387]	[0.0402]	[0.0444]	[0.0424]	[0.0408]	[0.0373]
Universal Health Coverage Index	0.00739***					
	[0.00128]					
Government effectiveness		0.0840***				
		[0.0191]				
Index of democratization			0.0031			
			[0.00219]			
Participation				0.00430**		
				[0.00197]		
Trust the national government (%)					0.00329***	
					[0.000906]	
State Capacity Index						0.108***
						[0.0259]
Constant	− 0.319*	0.0911	− 0.0853	− 0.175	− 0.157	0.00494
	[0.168]	[0.145]	[0.156]	[0.144]	[0.185]	[0.157]
Observations	86	85	86	86	69	79
R-squared	0.607	0.57	0.484	0.531	0.582	0.61

Macroregional effects included. Robust standard errors in brackets

*p < 0.1, **p < 0.05, ***p < 0.01

**Table 3:** Determinants of % of population fully vaccinated

Dep. var. % fully vaccinated	(1)	(2)	(3)	(4)	(5)	(6)
COVID deaths (July 2020)	0.000139*	0.000211**	0.000197**	0.000182**	0.000260***	0.000179*
	[0.0000792]	[0.0000849]	[0.0000873]	[0.0000887]	[0.0000920]	[0.0000906]
Number of vaccine types	0.0449***	0.0407***	0.0403***	0.0409***	0.0373***	0.0407***
	[0.00954]	[0.00956]	[0.0109]	[0.00970]	[0.0106]	[0.0102]
First vaccine-MRNA	− 0.0365	− 0.0124	0.0318	0.0381	0.0813**	− 0.0014
	[0.0362]	[0.0402]	[0.0376]	[0.0374]	[0.0395]	[0.0380]
Urban population (%)	0.00373***	0.00490***	0.00646***	0.00605***	0.00590***	0.00474***
	[0.00103]	[0.000888]	[0.00123]	[0.00121]	[0.00126]	[0.00106]
Federalist	− 0.043	− 0.0563	− 0.0627	− 0.0445	− 0.0404	− 0.0305
	[0.0394]	[0.0399]	[0.0458]	[0.0448]	[0.0401]	[0.0361]
Universal Health Coverage Index	0.00826***					
	[0.00114]					
Government effectiveness		0.101***				
		[0.0171]				
Index of democratization			0.00380*			
			[0.00234]			
Participation				0.00439**		
				[0.00214]		
Trust the national government (%)					0.00346***	
					[0.000908]	
State Capacity Index						0.128***
						[0.0233]
Constant	− 0.404***	0.0676	− 0.146	− 0.233*	− 0.214	− 0.03
	[0.150]	[0.123]	[0.140]	[0.130]	[0.161]	[0.133]
Observations	86	85	86	86	69	79
R-squared	0.639	0.615	0.491	0.531	0.59	0.655

Macroregional effects included. Robust standard errors in brackets

*p < 0.1, **p < 0.05, ***p < 0.01

**Table 4:** Determinants of % of population with additional dose

Dep. var. % additional dose	(1)	(2)	(3)	(4)	(5)	(6)
COVID deaths (July 2020)	0.000138*	0.000225***	0.000201**	0.000216**	0.000294***	0.000195**
	[0.0000789]	[0.0000839]	[0.0000929]	[0.0000985]	[0.0000889]	[0.0000782]
Number of vaccine types	0.0180**	0.0121	0.0115	0.0147*	0.00808	0.0104
	[0.00892]	[0.00831]	[0.00887]	[0.00845]	[0.0109]	[0.00969]
First vaccine-MRNA	0.00391	0.0358	0.0740*	0.0885**	0.116**	0.0361
	[0.0358]	[0.0427]	[0.0444]	[0.0440]	[0.0467]	[0.0400]
Urban population (%)	0.00398***	0.00529***	0.00690***	0.00687***	0.00569***	0.00451***
	[0.00121]	[0.00120]	[0.00148]	[0.00151]	[0.00154]	[0.00141]
Federalist country	− 0.0708	− 0.0837*	− 0.0958**	− 0.0823*	− 0.07	− 0.0586
	[0.0438]	[0.0459]	[0.0477]	[0.0504]	[0.0478]	[0.0442]
Universal Health Coverage Index	0.00896***					
	[0.00128]					
Government effectiveness		0.101***				
		[0.0188]				
Index of democratization			0.00381			
			[0.00243]			
Participation				0.00257		
				[0.00197]		
Trust the national government (%)					0.00366***	
					[0.00108]	
State Capacity Index						0.134***
						[0.0225]
Constant	− 0.703***	− 0.192*	− 0.404***	− 0.464***	− 0.462***	− 0.236**
	[0.0976]	[0.0999]	[0.113]	[0.121]	[0.112]	[0.116]
Observations	84	83	84	84	68	77
R-squared	0.705	0.654	0.559	0.556	0.618	0.686

Macroregional effects included. Robust standard errors in brackets

*p < 0.1, **p < 0.05, ***p < 0.01

### Institutional factors at the subnational level

Institutional quality varies across but also within countries, due to different constitutional, political and organizational relations between subnational governments and officials and the central government. To further expand on the idea that a federal country might organize vaccination programs differently from a unitary one, as considered in Sect. 4.1, additional institutional factors at the subnational level are now considered explicitly, with the aim of providing an initial answer to Hypothesis 2. The underlying mechanism that could be at play is related to the fact that, in different countries, subnational (regional, provincial or municipal, depending on each nation’s structure) may have different degrees of autonomy and authority over specific policies and their implementation and varying degrees of coordination with, and power from and over, central governments. A vaccination campaign thus could be organized, from a distributional and logistic point of view and with reference to implementation and definition of the target population in a more or less efficient manner depending on the degree of regional autonomy and authority and, more specifically, to how much independence subnational governments have in setting public policies. To verify if different regional institutional arrangements are indeed related to the success of the vaccination campaign and ascertain the sign of this relationship, two indicators are considered: the aggregate *Regional authority index* and its component related to policies, *Policy Autonomy*. The first ranges from 0 to 30, with values increasing with regional authority. The *Policy Autonomy* component instead ranges from 0 to 4, with higher values indicating countries where subnational units enjoy higher autonomy in setting policies and in the number of sectors for which this autonomy can be exercised. Results are presented in Table 5.^2^

**Table 5:** Institutional factors at the subnational level

	% Vaccinated	% Vaccinated	% Fully vaccinated	% Fully vaccinated	% Additional dose	% Additional dose
	(1)	(2)	(3)	(4)	(5)	(6)
COVID deaths	0.152	0.127	0.131	0.113	0.145	0.165*
	[0.0000999]	[0.0000958]	[0.0000941]	[0.0000910]	[0.000108]	[0.000115]
Number of vaccine types	0.281**	0.265**	0.219*	0.204*	0.068	0.07
	[0.0142]	[0.0135]	[0.0138]	[0.0130]	[0.0110]	[0.0108]
First vaccine-MRNA	0.044	0.057	0.098	0.112	0.205*	0.212*
	[0.0405]	[0.0391]	[0.0414]	[0.0402]	[0.0474]	[0.0472]
Urban population (%)	0.553***	0.552***	0.561***	0.559***	0.577***	0.575***
	[0.00134]	[0.00138]	[0.00131]	[0.00134]	[0.00160]	[0.00164]
Federalist country	− 0.187*	− 0.154*	− 0.223*	− 0.172*	− 0.285*	− 0.200*
	[0.0484]	[0.0378]	[0.0539]	[0.0429]	[0.0740]	[0.0637]
Regional authority index	0.261*		0.299**		0.232*	
	[0.00236]		[0.00251]		[0.00253]	
Policy autonomy		0.298***		0.314***		0.148
Observations	80	80	80	80	78	78
R-squared	0.413	0.432	0.404	0.42	0.511	0.504

Macroregional effects included. Robust standard errors in brackets

*p < 0.1, **p < 0.05, ***p < 0.01

When considering the aggregate *Regional authority index*, its estimated coefficient is positive and different from zero in all models, indicating that, at all stages of the process, countries with a higher degree of subnational authority in general are associated with higher outreach of the vaccination campaign, while the coefficient for *Federalism* is negative and statistically distinguishable from zero. Formal federalism as laid out in a country’s Constitution is negatively related to the percentage of population that had access and completed the vaccination program, while a more nuanced and comprehensive measure of regional autonomy is positively related. Similarly, when considering the *Policy Autonomy* component, for which similar considerations for this indicator and for federalism hold for people with one dose or that have completed the initial official program, but estimated coefficients are not distinguishable from zero for the additional dose.

An overarching reading of these results rests on the distinction between a federal state, where the central and subnational governments have a clear division of competencies, and different organizational forms of decentralization, which may encompass mutual cooperation and sharing of powers between different tiers of government. As suggested by Agnew (2022), in an in depth analysis of the US, it may not be an issue of federalism per se that has been detrimental in dealing with the COVID-19 crisis, but a dualist vision of federalism, particularly relevant today in the US, where the central and peripheral governments do not act cooperatively, leading also to potential conflicts among the subnational governments. Miller (2000), examining the fiscal aspects of immunization programs in the US, provides another potential mechanism that might lead to a less successful organization of vaccination campaigns in federations. His analysis points towards the imbalance between higher responsibility for federal States to provide immunization to the population and a contemporaneous decline in funding from the Central government for the same campaigns.

**Fig. 4:**
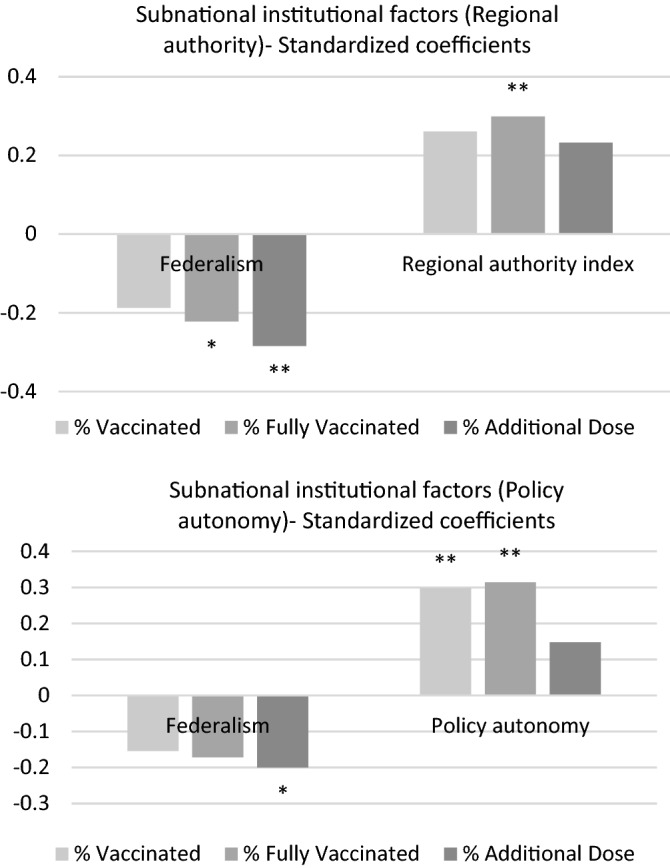
Standardized coefficients of subnational institutional factors

Figure 4 shows beta coefficients and supports the interpretation that what matters most in terms of a successful vaccination campaign is not only whether a country is organized as a federation or as a unitary country (which, if anything, is negatively related with the percentage of vaccinated population at different stages) but if its subnational components enjoy degrees of authority, specifically in terms of policy implementation.

### Spatial patterns of vaccination

**Fig. 5:**
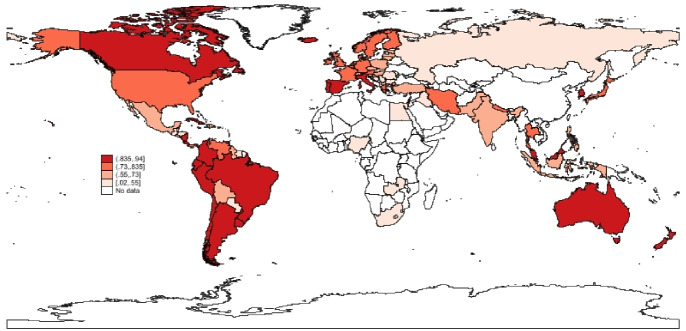
Vaccinated. *Source*: Author’s elaboration

**Fig. 6:**
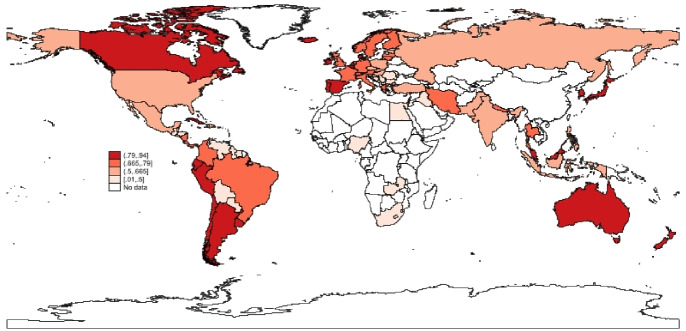
Fully vaccinated. *Source*: Author’s elaboration

**Fig. 7:**
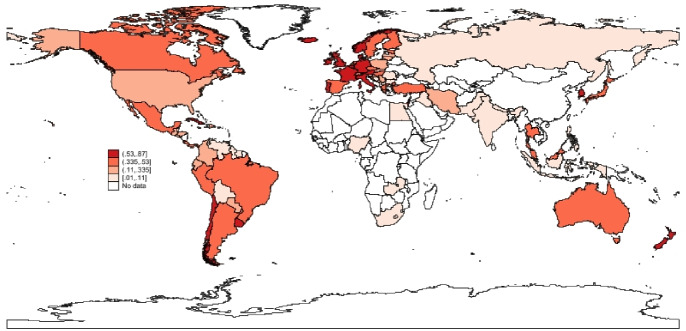
Additional dose. *Source*: Author’s elaboration

**Fig. 8:**
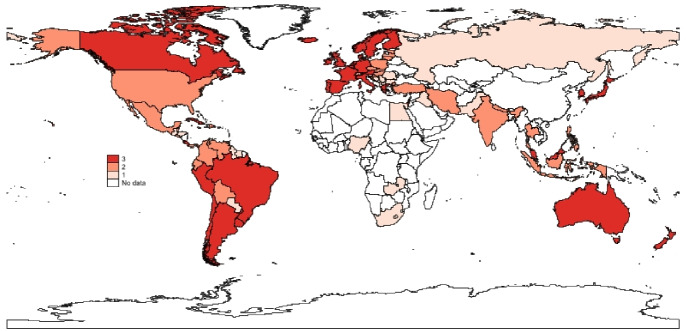
Cluster analysis. *Source*: Author’s elaboration

To complete the picture, a spatial analysis of the vaccination progress in the different countries in the sample is presented. In what follow, maps (Figs. 5, 6, 7) of the percentage of vaccinated population across stages and the results of a k-means cluster analysis on the same variables (Table 6 and Fig. 8) are provided to verify the existence of simple spatial patterns.

A first observation by inspecting the Figs. 5, 6 and 7 is that the percentage of population with at least one dose (Vaccinated) and those that have received the required number of doses (Fully Vaccinated) are generally highly correlated across countries. The same can not be claimed so clearly for Additional Doses, further corroborating the idea that different mechanisms are at play at different stages of the process (Hypothesis 1).  

The cluster analysis was performed to detect the presence of clusters of success of the vaccination campaign, by searching for clusters of countries with high, medium and low value for all stages. The k-means clustering method was adopted, with the aim of identifying three clusters of countries such that the distance between each observation is minimized with respect to the cluster’s mean. Considering the results of the formal cluster analysis (Fig. 8 and Table 6), countries with high percentages at all stages (Cluster 3) are mainly spatially clustered together in Western Europe and South America, with Canada and Australia as spatial outliers. Countries with lower, but still above 50% of vaccinated and fully vaccinated population (Cluster 2) are clustered in Eastern Europe, the northern part of South America and scattered in Asia. Countries in cluster 1, with very low levels of vaccinated population across all stages, do not seem to follow a specific spatial pattern. Tentative conclusions are related to a spatial correlation process which could be at the individual level, especially for additional doses, and at an institutional and legal level for vaccinated and fully vaccinated. More research is needed to further expand on these initial intuitions.

**Table 6:** Cluster analysis

	Mean	SD	Min	Max
*Cluster 1 (Obs. 34)*				
Vaccinated	0.844118	0.059804	0.74	0.94
Fully vaccinated	0.807941	0.064281	0.68	0.94
Additional dose	0.567941	0.097134	0.39	0.87
*Cluster 2 (Obs. 29)*				
Vaccinated	0.68931	0.088597	0.52	0.87
Fully vaccinated	0.622759	0.075019	0.5	0.8
Additional dose	0.256552	0.124277	0.02	0.44
*Cluster 3 (Obs. 24)*				
Vaccinated	0.426667	0.136212	0.1	0.61
Fully vaccinated	0.373333	0.127165	0.05	0.53
Additional dose	0.088333	0.065053	0.01	0.27

## Conclusions

By examining the correlation of external aspects on the percentage of vaccinated population at different stages of the COVID-19 vaccination campaign in a cross-section of countries, this paper contributes to the understanding of the success of universal vaccination campaigns. The focus is on supply side factors and institutional conditions, both at the national and subnational level, factors that can be the object of specific public policy initiatives to increase the percentage of vaccinated population.

The main results of the empirical analysis thus suggest a series of policy implications. As countries with higher urban density have higher vaccination rates at all stages, a target for specific communication initiatives and improved logistics are non-urban areas. Reliance on a single vaccine type or producer may hamper the success of the vaccination campaign, suggesting that authorities should try to secure vaccines from a variety of sources to avoid bottlenecks and shortage issues.

Focusing on the national-level institutional variables, the importance of a well-functioning healthcare system suggests focusing on reforms aimed at strengthening the core of the provision and distribution of health services. Investing in increasing government effectiveness and state capacity, not only related to the health sector, appears another potential avenue related to vaccination success. Finally, monitoring the levels of social capital and promoting actions aimed at increasing trust and cohesiveness among the population are also relevant actions to be considered.

Considering instead institutional factors considered at the subnational level, the organization of the country as federalist is negatively associated with the vaccination program, although this result is not particularly robust across empirical specifications. Subnational authority, especially in the form of subnational governments’ autonomy in setting and implementing policies is instead significantly related to more vaccinated population at all stages of the process, suggesting that countries where subnational governments enjoy more freedom in setting and implementing policies may be better equipped to align the policy response to a pandemic to the local needs. More research is however needed to understand other forms of decentralization and the interplay between different tiers of government, especially considering the role of federations and the degree of cooperation between central and peripheral governments.

The results of the present empirical analysis, based on a cross-section of country level for COVID-19 vaccination data could be further refined and strengthened by considering the time series dimension and by looking at immunization programs for other diseases. This would allow overcoming the potential limitations of the present study related to the aggregate nature of the data for a specific immunization program.

Additionally, from a social planner's perspective, the supply-side factors could be singled out and examined more in depth, in order to provide guidance on the organization of successful vaccination programs, also considering further spatially disaggregated data.

With the emergence of new variants, in the short run, and the possibility of new pandemics in the longer run, a clearer understanding of the determinants of the pace, success and adhesion to a vaccination program, is crucial, and this paper aims at being an initial contribution to this strand of research.

## Appendix

See Tables 7 and 8.

**Table 7:** Data sources and variable description

Variable	Description	Source	Date
% Vaccinated	Cumulative persons vaccinated with at least one dose per 100 population	WHO	April 2022
% Fully vaccinated	Cumulative number of persons fully vaccinated per 100 population	WHO	April 2022
% Additional dose	Persons received booster or additional dose per 100 population	WHO	April 2022
COVID deaths (July 2020)	cumulated deaths due to COVID-19 per 1,000,000 inhabitants	OWID	July 2020
Number of vaccine types available	Number of vaccine types used per country	WHO	April 2022
First vaccine-MRNA	1 = first administered vaccine MRNA type, 0 = first administered vaccine other technology	author's elaboration based on WHO data	Different dates
Urban population (%)	Urban population (% of total population)	United Nations	2019
Federalist country	1 = federalist country, 0 = unitary country	Forum of federations	2022
Universal Health Coverage Index	Coverage of essential health services. The indicator is an index reported on a unitless scale of 0 to 100, which is computed as the geometric mean of 14 tracer indicators of health service coverage. The tracer indicators are as follows, organized by four components of service coverage: (1) Reproductive, maternal, newborn and child health. (2) Infectious diseases. (3) Noncommunicable diseases. (4) Service capacity and access	WHO	2019
Government effectiveness	Combines into a single grouping responses on the quality of public service provision, the quality of the bureaucracy, the competence of civil servants, the independence of the civil service from political pressures, and the credibility of the government’s commitment to policies. The main focus of this index is on “inputs” required for the government to be able to produce and implement good policies and deliver public goods	Worldwide Governance Indicators (retrieved from the QoG dataset, Teorell et al. 2022)	2020
Index of Democratization	The index of democratization is formed by multiplying the competition (electoral success of smaller parties) and the participation (voting turnout in each election) variables and then dividing the outcome by 100	Finnish Social Science Data Archive (retrieved from QoG dataset, originally by Vanhanen (2019))	2018
Participation	The political participation variable portrays the voting turnout in each election, and is calculated as the percentage of the total population who actually voted in the election	Finnish Social Science Data Archive (retrieved from QoG dataset, originally by Vanhanen (2019))	2018
Trust the national government (%)	Trust the national government in this country (%)	United Nations	2020
Hanson & Sigman State Capacity Index	Three dimensions of state capacity that their estimate relies on are extractive capacity, coercive capacity, and administrative capacity	Hanson and Sigman (2021), retrieved from QoG dataset	2015
Regional Authority Index	Regional authority index, which is the sum of the authority exercised by a regional government over those who live in the region and the authority exercised in the country as a whole	Hooghe et al. (2016)	2018
Policy autonomy	The range and sectors of policies for which a regional government is responsible	Hooghe et al. (2016)	2018

**Table 8:** List of countries

Albania	Germany	Nicaragua	
Argentina	Greece	Nigeria	
Australia	Guatemala	Norway	
Austria	Guyana	Pakistan	
Bahamas	Haiti	Panama	
Barbados	Honduras	Paraguay	
Belgium	Hungary	Peru	
Belize	Iceland	Philippines	
Bolivia	India	Poland	
Bosnia and Herzegovina	Indonesia	Portugal	
Brazil	Iran	Romania	
Brunei	Iraq	Russian Federation	
Bulgaria	Ireland	Serbia	
Canada	Israel	Singapore	
Chile	Italy	Slovak Republic	
Colombia	Jamaica	Slovenia	
Costa Rica	Japan	South Africa	
Croatia	Korea	Spain	
Cuba	Kosovo	Suriname	
Cyprus	Latvia	Sweden	
Czech Republic	Lithuania	Switzerland	
Denmark	Luxembourg	Thailand	
Dominican Republic	Macedonia (FYR)	Trinidad and Tobago	
East Timor	Malaysia	Turkey	
Ecuador	Malta	United Kingdom	
Egypt	Mexico	United States	
El Salvador	Montenegro	Uruguay	
Estonia	Nepal	Venezuela	
Finland	Netherlands	Zambia	
France	New Zealand		

## Footnotes

1 Different measures of human capital have been added to the specification. The associated coefficient is positive and the one estimated for urban population remains positive but decreases in magnitude. Results available upon request.

2 Since the estimation results are substantially the same as in the previous sections, they will not be discussed further.
